# Unusual Confluence: Exploring the Association of Biliary Atresia, Wilson Disease, and Iron Overload

**DOI:** 10.14309/crj.0000000000001500

**Published:** 2024-10-18

**Authors:** Shivangini Duggal, Samantha Meza-Rodriguez, Saqib Shahid, Marc Zuckerman, Jorge Chiquie Borges

**Affiliations:** 1 Department of Internal Medicine, Texas Tech University Health Sciences Center, El Paso, TX; 2 Texas Tech University Health Sciences Center, El Paso, Paul L. Foster School of Medicine, El Paso, TX; 3 Division of Gastroenterology, Texas Tech University Health Sciences Center, El Paso, TX; 4 Division of Cardiology, Texas Tech University Health Sciences Center, El Paso, TX

**Keywords:** Biliary atresia, Wilson disease, iron overload

## Abstract

The case involves a 33-year-old man with biliary atresia, Wilson disease (WD), and iron overload. Biliary atresia, a cholangiodestructive disease, leads to cirrhosis if untreated. WD, caused by ATP7B gene mutations, results in copper accumulation affecting the liver and brain. Iron overload can be seen in cases of WD and with hereditary hemochromatosis gene mutations. The patient's concurrent presentation of these conditions poses a unique clinical challenge. Elevated iron levels may worsen WD outcomes. A detailed history and physical examination, genetic testing, and close follow-up are crucial. The case highlights the need for increased awareness and vigilant monitoring of patients with overlapping liver diseases.

## INTRODUCTION

Biliary atresia (BA) is a cholangiodestructive disease that affects the biliary tract, leading to cirrhosis, liver failure, and potentially death, particularly prevalent in Asian countries. Up to 10% of cases may involve congenital anomalies such as splenic or venous malformations. The atresia of extrahepatic and intrahepatic bile ducts in BA obstructs the transport of conjugated bilirubin to the intestine, causing inflammation and cholestatic cirrhosis. Genes such as Adducin 3, Glypican 1, and ADP ribosylation factor 6 have been associated with BA. Early detection within the first 3 months allows for Kasai hepatoportoenterostomy, offering a chance of survival for half of the affected children after 2 years.^1–4^ Wilson disease (WD) is an autosomal recessive disorder caused by homozygous or compound heterozygous mutations in the ATP7B gene, which facilitates the transport of copper into bile for the synthesis of ceruloplasmin (CP).^5^ This dysfunction leads to copper accumulation in other organs, primarily the liver and the brain, resulting in pathognomonic symptoms.^6^ Copper and iron metabolism are interrelated, with copper influencing iron homeostasis positively, while iron antagonizes copper metabolism.^7^ Hereditary hemochromatosis (HH) is defined by systemic iron overload (IO) due to genetic mutations of the hereditary hemochromatosis gene (HFE), causing reduced levels of hepcidin or hepcidin-ferroportin binding.^8^ The HFE genetic test identifies the most common inherited defect in Whites predisposing to HFE-related HH, that is, p.Cys282Tyr homozygosity. p.Cys282Tyr and p.His63Asp heterozygosity have minimal or no clinical penetrance and is not considered diagnostic for HH but is associated with mild-to-moderate IO.^6,9^

## CASE REPORT

This case report describes a 33-year-old man with a history of BA who underwent the Kasai procedure 2 months age. He presented with complaints of abdominal pain, fatigue, dyspnea on exertion, blurry vision, and bilateral lower extremity swelling. Further investigation revealed liver cirrhosis due to WD. The patient had multiple hospital visits for hepatic encephalopathy and abdominal tenderness while on active military duty. Liver biopsy confirmed WD with liver copper levels of 300 ncg (more than 250 ncg).

On admission, he had scleral icterus, jaundiced skin with excoriations, and spider angiomas were observed on the chest and abdomen. The slit-lamp examination revealed Kayser-Fleischer rings. He had a 14 cm horizontal laparotomy scar in his right upper quadrant and diffuse abdominal tenderness. The significant laboratory results are listed below in Table 1. His imaging revealed liver cysts, ruling out hepatocellular carcinoma. His serum CP was low normal at 26 mg/dL, and urine copper levels were elevated at 275 mcg/24 hr. His Leipzig score was greater than 4, establishing a high suspicion of WD. Tests for HH showed positive results for heterozygous mutations of the HFE gene (H63D), and he was negative for the C282Y pathogenic variant, indicating his carrier status. His ferritin levels were very elevated, greater than 2,000 ng/mL, but he had low transferrin levels and transferrin saturation. His new Wilson Index was 13 (indicating greater mortality without transplant), Model for End-Stage Liver Disease was 40 points (with a 71.3% estimated 3-month mortality), and Child-Pugh was 13 points (with a life expectancy of 1–3 years). The patient has since been readmitted to our facility multiple times for the management of decompensated cirrhosis. He underwent liver transplantation and is currently doing well.

**Table 1.** Laboratory workup on admission (on admission, his laboratory results revealed)

Tests	Results	Normal range
White blood count (WBC)	5.80 × 103/μL	4.50–11.03/μL
Red blood count (RBC)	2.21 × 106/μL L	3.50–5.50/μL
Hemoglobin	8.3 g/dL L	12.0–15.0 g/dL
Platelets	75 × 103/μL L	150–450 × 103/μL
Sodium, serum	130 mmol/L	135–145 mmol/L
Potassium, serum	3.7 mmol/L	3.5–5.1 mmol/L
Chloride, serum	107 mmol/L	98–107 mmol/L
Bicarbonate	17 mmol/L	22–30 mmol/L
Glucose	90 mg/dL	74–106 mg/dL
Blood urea nitrogen, serum	34 mg/dL H	7–17 mg/dL
Magnesium, serum	2.5 mg/dL H	1.6–2.3 mg/dL
Creatinine	2.10 mg/dL H	0.70–1.3 mg/dL
Albumin	2.0 g/dL L	3.4–5.4 g/dL
Total bilirubin	24.7 mg/dL H	0.1–1.2 mg/dL
Bilirubin direct	22.4 mg/dL H	<0.3 mg/dL
AST (GOT)	168 IU/L H	14–20 IU/L
Alkaline phosphatase	236 IU/L	44–147 IU/L
ALT (GPT)	96 IU/L H	29–33 IU/L
Lipase	154 U/L	0–160 U/L
Amylase	52.0 U/L	40–140 U/L
Iron serum	87 mcg/dL	60–170 mcg/dL
Transferrin	<80 mg/dL L	215–380 mg/dL
TIBC	112 mcg/dL L	240–450 mcg/dL
Ferritin	2,630 ng/mL H	12–300 ng/mL
Alpha fetoprotein	1.06 ng/mL	5–10 ng/mL
ANA	Negative	Negative
HBSAG	Negative	Negative
B core	Negative	Negative
Hepatitis A IgM	Negative	Negative
Hepatitis C Virus	Negative	Negative
Ceruloplasmin	26 mg/dL Low N	18–36 mg/dL
Urine copper, 24 hr	275 mcg/24 hr H	15–60 mcg/24 hr
Mitochondrial M2 Ab	Negative	Negative
Alpha-1 Antitrypsin phenotype	PI*MM	MM
Alpha-1 Antitrypsin QN	150 mg/dL	83–199 mg/dL
Smith antibody	Negative	Negative
Haptoglobin	51 mg/dL	43–212 mg/dL

ANA, antinuclear antibodies; AST (GOT), aspartate aminotransferase (glutamic oxaloacetic transaminase); ALT (GPT), alanine aminotransferase (glutamic pyruvic transaminase); HBSAG, hepatitis B surface antigen; IgM, immunoglobulin M; PI*MM, protease inhibitor homozygous for M allele; QN, quantitative; TIBC, total iron binding capacity.

## DISCUSSION

Our case provides the first documented instance of clinical correlation between 3 distinct liver diseases, namely BA, WD, and HH.

**Figure 1.**
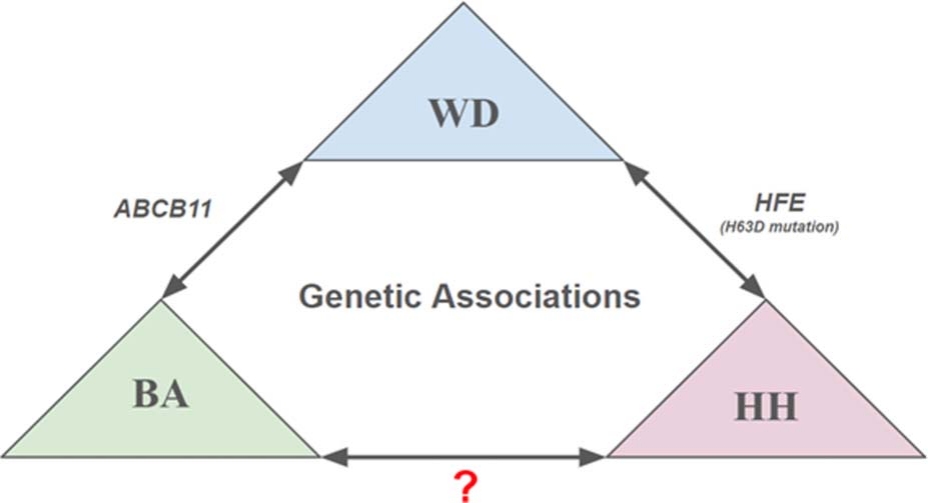
As discussed, certain cases raise the possibility of genetic association between other hepatobiliary disorders and Wilson disease. In the case from Hoa et al, the patient was found to have a homozygous variant of the ABCB11 gene concurrent with 2 heterozygous mutations in ATPase copper transporting beta. Abuzetun et al present a scenario where a patient with the H63D mutation of the HFE gene suffers from Wilson disease in addition to hereditary hemochromatosis. These findings reasonably demonstrate an association between biliary atresia, hereditary hemochromatosis, and Wilson disease. It remains to be seen however, if a similar association exists between biliary atresia and hemochromatosis. ABCB11, ATP-binding cassette, sub-family B member 11; HFE, hereditary hemochromatosis gene.

WD typically manifests with isolated liver disease, and diagnosis involves evaluating Kayser-Fleischer rings, serum CP levels, urine copper levels, liver biopsy, and genetic testing. Neuroimaging is recommended for patients with neurological symptoms.^10^ Nguyen Pham Anh Hoa et al reported a case of an 8-year-old Vietnamese girl who suffered from BA as an infant and later was diagnosed with WD, presenting severe symptoms of acute liver failure^11^. The authors identified a homozygous variant in the ATP-binding cassette, sub-family B member 11 gene as a precipitator of bile accumulation, responsible for inflammation and destruction of biliary ducts resulting in BA, particularly in the Vietnamese population.^11^ The case presented here is unique, involving a Polynesian male with coexisting BA and WD, a scenario not reported in the literature (Figure 1).

Extremely elevated iron levels have sporadically been described in patients with WD, and phenotypic variability with increased ferritin levels in male patients compared with female patients has been proposed in the background of HFE mutations.^12,13^ Various copper-dependent enzymes are associated with iron metabolism, the most notable being CP, an iron ferroxidase that also serves as the primary copper transporter in the blood. In WD, reduced incorporation of copper into apoceruloplasmin by ATP7B leads to rapid degradation, resulting in decreased cellular secretion of CP and lower serum CP concentrations. This also leads to reduced CP oxidase activity. Consequently, CP-facilitated basolateral iron transport may be impaired in WD. Eventually, this impairment could lead to cellular iron trapping and a subsequently reduced bioavailability of iron.^13^ Abuzetun et al reported a rare case of HH and WD coexisting, describing similar heterozygous mutations of HH (H63D), while Gromadzka et al conducted a study on the effect of homeostatic iron regulatory protein gene mutations on WD manifestations, finding that 31% of their subjects with WD also had coexisting heterozygous HFE 63HD mutations, with male HFE 63HD heterozygotes showing symptoms of WD slightly earlier than HFE 63HH homozygotes; our patient exhibits similar findings with a heterozygous HFE 63HD mutation and biopsy-confirmed WD.^14,15^ As the role of HFE mutations in HH is controversial, IO in our patient may be secondary to WD rather than hemochromatosis. Elevated iron levels may lead to worse outcomes in patients with WD due to iron accumulation in the liver or brain (putamen and globus pallidus), worsening the overall prognosis.^15–17^ A lower threshold of Model for End-Stage Liver Disease scores for liver transplant consideration is required in patients with WD and coexisting HH mutations.

WD requires a thorough evaluation involving history, physical examination, and various tests for a definitive diagnosis. For children diagnosed with BA, close follow-up and genetic workup for liver diseases such as WD and HH are recommended to prevent later complications. Concurrent liver diseases can worsen the prognosis, leading to rapid liver failure in young individuals. Clinicians are urged to remain vigilant for such associations, as timely treatment is crucial to avoid serious consequences for the patient.

## DISCLOSURES

Author contributions: This paper was conceptualized by S. Duggal, S. Meza-Rodriguez, and S. Shahid. The investigation and review of relevant data and articles done by S. Duggal, S. Meza-Rodriguez, and S. Shahid. Case analysis was done by S. Duggal, SMR, and S. Shahid. The original draft was written by S. Duggal, S. Meza-Rodriguez, and S. Shahid. Review and editing were done by M Zuckerman and JC Borges. All authors discussed the findings described in the case and approved the final manuscript. JC Borges is article guarantor.

Financial disclosure: None to report.

Informed consent was obtained for this case report.
